# Low-Temperature Molten Salt Synthesis and the Characterisation of Submicron-Sized Al_8_B_4_C_7_ Powder

**DOI:** 10.3390/ma13010070

**Published:** 2019-12-22

**Authors:** Cheng Liu, Xueyin Liu, Zhaoping Hou, Quanli Jia, Benjun Cheng, Shaowei Zhang

**Affiliations:** 1 College of Engineering, Mathematics and Physical Sciences, University of Exeter, Exeter EX4 4QF, UK; 2 The State Key Laboratory of Refractories and Metallurgy, Wuhan University of Science and Technology, Wuhan 430081, China; kklldliu@163.com; 3 College of Materials Science and Engineering, Taiyuan University of Technology, Taiyuan 030024, China; houzhaoping@tyut.edu.cn; 4 Henan Key Laboratory of High Temperature Functional Ceramics, Zhengzhou University, Zhengzhou 450052, China; jiaquanli@zzu.edu.cn; 5 School of Energy Science and Engineering, Central South University, Changsha 410205, China

**Keywords:** Al_8_B_4_C_7_, molten salt synthesis, low temperature, carbon black, B_4_C

## Abstract

Submicron-sized (~200 nm) aluminium boron carbide (Al_8_B_4_C_7_) particles were synthesised from Al, B_4_C and carbon black raw materials in a molten NaCl-based salt at a relatively low temperature. The effects of the salt type/assembly and the firing temperature on the synthesis process were examined, and the relevant reaction mechanisms discussed. The molten salt played an important role in the Al_8_B_4_C_7_ formation process. By using a combined salt of 95%NaCl + 5%NaF, an effective liquid reaction medium was formed, greatly facilitating the Al_8_B_4_C_7_ formation. As a result, essentially phase-pure Al_8_B_4_C_7_ was obtained after 6 h of firing at 1250 °C. This temperature was 350–550 °C lower than that required by the conventional direct reaction and thermal reduction methods.

## 1. Introduction

Like their binary counterparts, ternary carbides are also an important class of high-performance non-oxide ceramics that have attracted great research interest, especially during the past decade. Among them, aluminium boron carbide (Al_8_B_4_C_7_ or Al_3_BC_3_) is regarded as a promising candidate material potentially applicable to a variety of important areas, such as high-temperature structural ceramics, as an antioxidant for carbon-containing refractories [1,2,3,4,5], as a sintering agent for SiC, ZrB_2_ and B_4_C materials [6,7,8,9,10], and as an absorber for new-generation nuclear reactors [11]. This is because of its many superior properties, including high hardness (15.2 GPa), high melting point (1900 °C), relatively low density (2.69 g/cm^3^) and low thermal expansion coefficient (6.67 × 10^−6^/K), high thermal conductivity (29.2 W/m/K), and good oxidation/corrosion resistance [1,12,13,14].

To fabricate high-performance Al_8_B_4_C_7_-based bulk ceramics, high-quality Al_8_B_4_C_7_ powder often needs to be used. In this regard, several synthesis methods/techniques have been developed to date, among which the thermal reduction and direct reaction methods have been investigated most extensively. In the former, Al or carbon (C) is often used as a reducing agent [15,16,17,18], so inexpensive and readily available boron-containing oxides can be used as a boron source to replace the much more expensive element boron (B) or B_4_C. However, a high synthesis temperature (1700–1800 °C) is required to complete the formation reaction. Furthermore, some by-products/intermediate phases such as Al_2_O_3_, Al_4_O_4_C and Al_2_OC often remain in the final product powder. In addition, the product particles generally have relatively large sizes and suffer from heavy agglomeration. For example, Zhu et al. [15] and Deng et al. [16] prepared Al_8_B_4_C_7_ powder containing secondary phases of Al_2_O_3_, Al_2_OC and Al_4_O_4_C at 1700 °C by using B_2_O_3_ (or Na_2_B_4_O_7_·10H_2_O), Al and C as raw materials. By using similar raw materials and a higher temperature (1800 °C), Cui et al. [17] prepared hexagonal micro-platelets of Al_8_B_4_C_7_ containing minor Al_2_OC. On the other hand, Lee et al. [18] prepared Al_3_BC_3_ via a complex route using Al(OH)_3_, B_2_O_3_ and phenolic resin as raw materials. Despite the use of phenolic resin instead of solid C powder, the synthesis temperature still remained as high as 1725 °C.

In contrast to the thermal reduction method, no reducing agent is required for the direct reaction method. The raw material assemblies commonly used by this method include: (1) Al_4_C_3_ and B_4_C; (2) Al, B and C; and (3) Al, B_4_C and C. Unfortunately, this method also suffers from similar drawbacks to those of the thermal reduction method, i.e., high synthesis temperature (1600–1800 °C) [11,13,14,19,20,21], and relatively large sizes of product particles with heavy agglomeration among them. For example, Inoue et al. [22] synthesised Al_8_B_4_C_7_ powder via a respectively direct solid–solid reaction between Al_4_C_3_ and B_4_C at 1800 °C, and double stage reactions between Al, B and C initially at 1400 °C and then at 1830 °C. Several other researchers, e.g., Gao et al. [21], Hashimoto et al. [14] and Wang et al. [13], also synthesised Al_8_B_4_C_7_ powder at 1600–1800 °C by using Al, B_4_C and C as raw materials.

To overcome the drawbacks of the two main synthesis techniques stated above, it is necessary to develop alternative techniques. As a response to this, in the present work, a molten salt synthesis (MSS) method, used previously to prepare oxide and binary carbide powders [23,24,25], was further developed and extended to synthesise high-quality submicron-sized Al_8_B_4_C_7_ powder at a much lower temperature, from Al, B_4_C and C starting materials. As-prepared Al_8_B_4_C_7_ powder was characterised, and the effects of key processing factors such as firing temperature and salt type/assembly on MSS were investigated. Based on the experimental results, the synthesis/formation mechanism of Al_8_B_4_C_7_ was discussed.

## 2. Experimental Procedure

### 2.1. Raw Materials

Al (99.7% pure, <25 micron), B_4_C (99.98% pure), amorphous B (95% pure) and C (carbon black, ≥99% pure, <250 nm) powders were used as raw materials, and NaCl (≥99%) and NaF (≥98%) were used to form the desired liquid reaction medium. They were all purchased from Sigma-Aldrich (Gillingham, UK).

### 2.2. Sample Preparation

Al, B_4_C and C were mixed in the stoichiometric molar ratios of 8:1:6 (1.35:0.35:0.53 g in a powder batch) corresponding to Equation (1), and then they were further combined with 20 g binary salt of 95%NaCl + 5%NaF in an agate mortar. The mixed powder batch was contained in a graphite crucible covered with a graphite lid, and then it was placed in an alumina tube furnace protected by flowing argon (Ar). The furnace was heated to a target temperature between 1100 and 1250 °C (at 5 °C/min to 1000 °C, then 3 °C/min to 1200 °C and finally 1 °C/min to the target temperature) and held at the temperature for 6 h.

8Al + B_4_C + 6C = Al_8_B_4_C_7_ (1)

To study the effects of salt type/assembly on the formation of Al_8_B_4_C_7_, two other types of salts (NaCl, and 97.5%NaCl + 2.5%NaF) were used, as well as the binary salt NaCl-NaF stated above, to form the reaction media. They were then compared.

In addition, to assist in clarifying the relevant reaction/formation mechanisms, the following supplementary experiment was also carried out, and the resultant samples were similarly characterised (Section 2.3 below). In the first test, Al and B_4_C (1.35 and 0.35 g) in the molar ratio of 8:1 (referred to as Al-B_4_C sample) were heated in 20 g of 95%NaCl + 5%NaF at 1250 °C for 6 h. The reacted mass was further combined with 0.53 g C (so the molar ratio of Al:B_4_C:C = 8:1:6) and reheated at 1250 °C for 6 h in the identical salt. In the second test, Al_4_C_3_ (prepared via the reaction of stoichiometric amounts of Al and C in 20 g of 95%NaCl + 5%NaF at 1150 °C for 6 h) was combined with B and C (the molar ratio of Al/B_4_C/C = 8:1:6) (referred to as Al_4_C_3_-B-C sample) and fired at 1250 °C for 6 h.

Some of the samples after firing were placed immediately in a desiccator to avoid the hydration of Al_4_C_3_ in them prior to characterisation, whereas the other fired samples were subjected to repeated hot water washing to leach out the residual salt, followed by overnight oven-drying at 100 °C.

### 2.3. Sample Characterisation

Phases in fired samples were identified by powder X-ray diffraction (XRD) analysis (Bruker D8 advance reflection diffractometer, Karlsruhe, Germany). The diffractometer was operated at 40 mA and 40 kV using Ni-filtered CuKa radiation. The scan rate was 2.4° (2θ)/min with a step size of 0.04°. The ICDD cards used for identification were Al_8_B_4_C_7_ (35-1216), Al_3_BC_3_ (88-1267), Al_4_C_3_ (35-0799), AlOOH (21-1307), Al (65-2869), Al_2_O_3_ (46-1212), Al_3_BC (50-1470), AlB_2_ (65-9698) and NaCl (05-0628). The microstructure and morphology of the as-prepared product powder were observed using a scanning electron microscope (SEM Nova Nanolab 600, FEI Company, Hillsboro, OR, USA) and a JEM 2100 transmission electron microscope (TEM, 200 kV).

## 3. Results and Preliminary Discussion

### 3.1. Effect of Firing Temperature on the Formation of Al_8_B_4_C_7_

**Figure 1:**
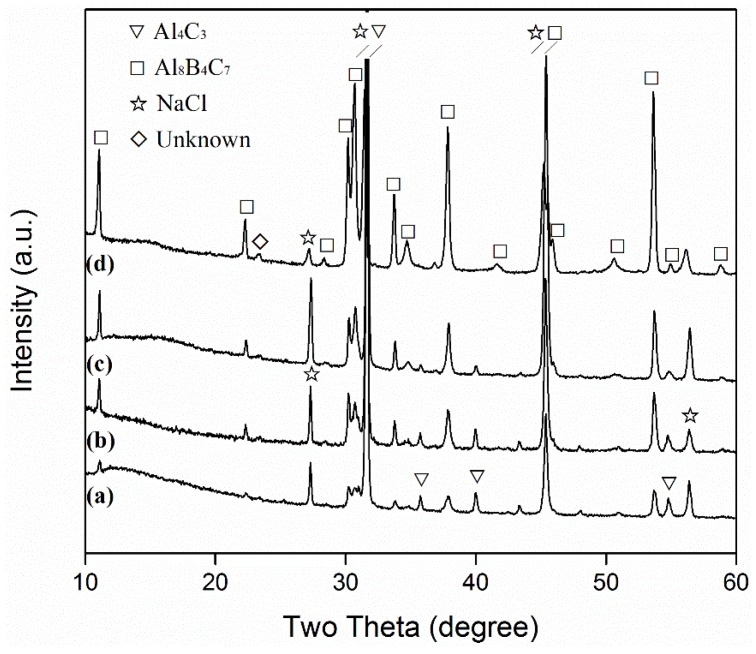
X-ray diffraction (XRD) patterns of samples resulting from 6 h of firing in 95%NaCl + 5%NaF salt at: (**a**) 1100, (**b**) 1150, (**c**) 1200, and (**d**) 1250 °C, respectively (prior to water washing).

**Figure 2:**
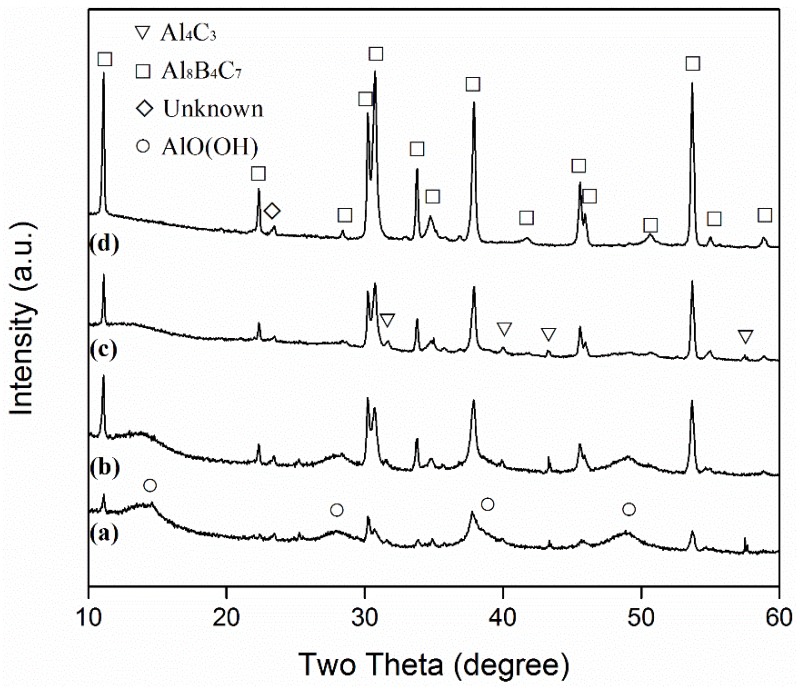
XRD patterns of samples resulting from 6 h of firing at: (**a**) 1100, (**b**) 1150, (**c**) 1200, and (**d**) 1250 °C, respectively (after water washing).

Figure 1 and Figure 2 show XRD patterns of samples resulting from 6 h of firing at different temperatures in 95%NaCl + 5%NaF, before and after water washing, respectively. The formation of Al_8_B_4_C_7_ was already evident after 6 h at 1100 °C, but the intermediate phase of Al_4_C_3_ was also detected (Figure 1a). With an increase in temperature to 1150 and then 1200 °C, Al_8_B_4_C_7_ increased, whereas Al_4_C_3_ decreased (Figure 1b,c). Upon increasing the temperature to 1250 °C, Al_4_C_3_ disappeared, and only Al_8_B_4_C_7_ was identified (though a minor peak from an unknown phase appeared at 2θ = 23.3°), i.e., essentially phase-pure Al_8_B_4_C_7_ was formed (Figure 1d). AlOOH was detected in some of the washed samples (Figure 2a–c) due to the partial hydration of Al_4_C_3_ in the original fired samples (Figure 1a–c) during the repeated water-washing process (Equation (2)). As shown in Figure 2, AlOOH, i.e., Al_4_C_3_, decreased with the increase in the corresponding firing temperature. It disappeared upon increasing the firing temperature to 1250 °C, verifying the completion of the formation reaction at this temperature (Figure 2d).

Al_4_C_3_ + 8H_2_O = 4AlOOH + 3CH_4_ (2)

### 3.2. Supplementary Experiment for Mechanism Clarification

**Figure 3:**
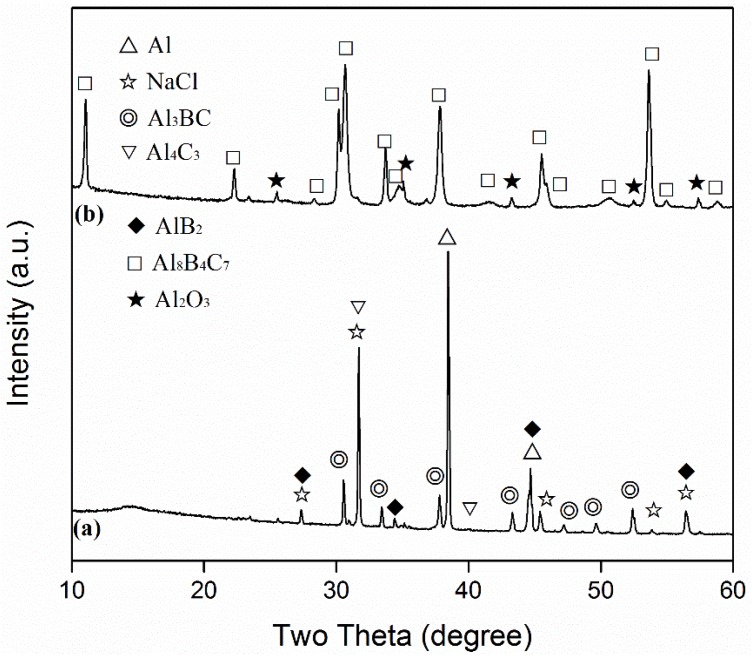
XRD patterns of: (**a**) the Al-B_4_C sample after 6 h of firing in 95%NaCl + 5%NaF at 1250 °C, and (**b**) the sample resulting from further firing of (**a**) with C in 95%NaCl + 5%NaF at 1250 °C for 6 h.

**Figure 4:**
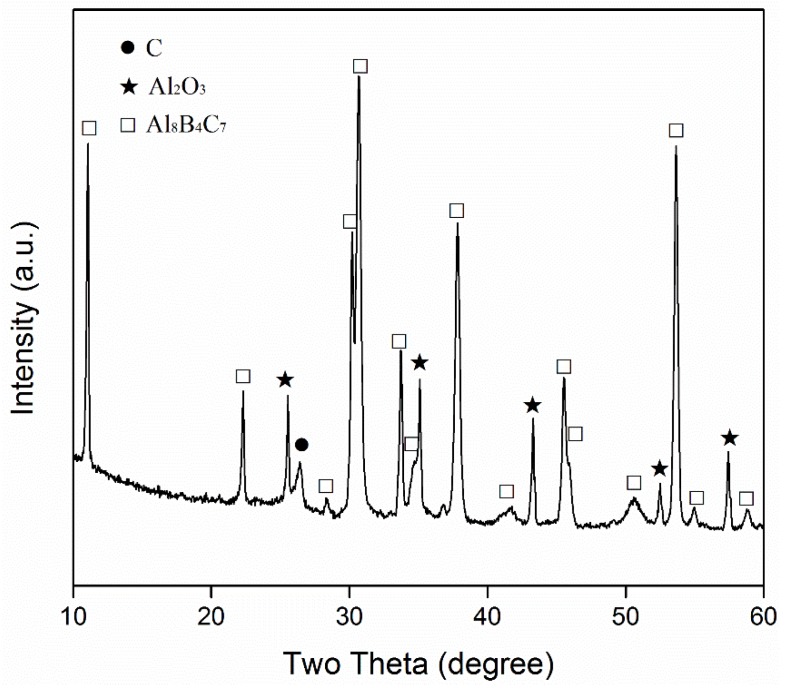
XRD pattern of the Al_4_C_3_-B-C sample after 6 h of firing in 95%NaCl + 5%NaF at 1250 °C.

Shown in Figure 3 are the XRD patterns of the Al-B_4_C sample after the first-stage firing, and the patterns after subsequent re-firing with C in the 95%NaCl + 5%NaF salt. After the first-stage firing (Figure 3a), Al_3_BC was formed as the main phase, along with some AlB_2_. However, after the second-stage firing with C, Al_8_B_4_C_7_ became the primary phase (Figure 3b), suggesting that the Al_3_BC that formed in the sample after the first-stage firing was converted into Al_8_B_4_C_7_. Figure 4 further presents the XRD pattern of the Al_4_C_3_-B-C sample after 6 h of firing in the 95%NaCl + 5%NaF salt at 1250 °C, revealing the formation of the primary phase of Al_8_B_4_C_7_, as well as minor residual C and Al_2_O_3_. The minor Al_2_O_3_ detected in this case (also in Figure 3b) was likely a result of the decomposition of AlOOH formed from the quick hydration of Al_4_C_3_ by the moisture in the atmosphere during the sample processing.

### 3.3. Effect of Salt Type/Assembly on the Formation of Al_8_B_4_C_7_

**Figure 5:**
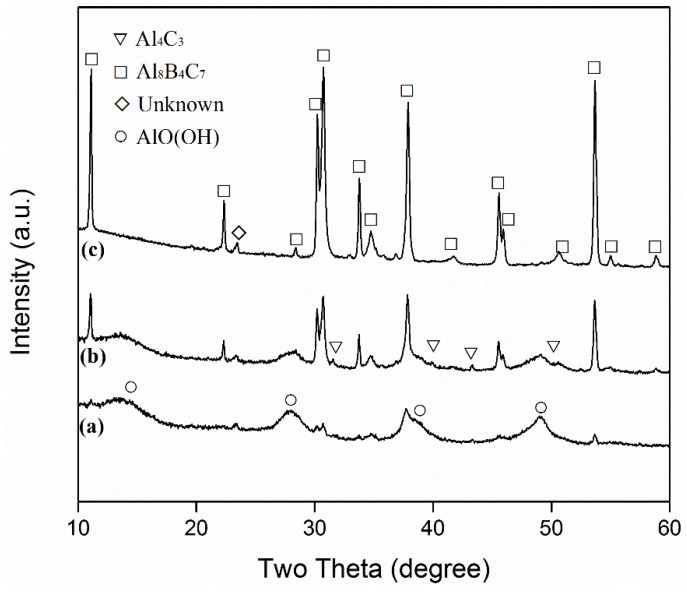
XRD patterns of samples resulting from 6 h of firing at 1250 °C in 20 g of (**a**) NaCl, (**b**) 97.5%NaCl+2.5%NaF, and (**c**) 95%NaCl + 5%NaF, respectively (after water washing).

Figure 5 demonstrates the effect of salt type/assembly on the Al_8_B_4_C_7_ formation. In the case of using NaCl (Figure 5a), only minor Al_8_B_4_C_7_ was formed, but large amounts of AlOOH were detected in the water-washed sample, indicating the presence of large amounts of intermediate Al_4_C_3_ in the original fired sample. This implied the limited accelerating effect of NaCl on the Al_8_B_4_C_7_ formation. However, when small amounts (0.5 g, i.e., 2.5%) of NaF were combined with NaCl, Al_8_B_4_C_7_ became the main phase, although some AlOOH (i.e., Al_4_C_3_ in the original fired sample) was still detected (Figure 5b). This indicated the great accelerating effect of the NaF addition on the overall synthesis process. Upon further increasing the NaF amount to 1 g (i.e., 5%), AlOOH (i.e., Al_4_C_3_) disappeared and essentially phase-pure Al_8_B_4_C_7_ was formed (Figure 5c). The above results indicated that the optimal salt type/assembly in the present work was 95%NaCl + 5%NaF.

### 3.4. Microstructure of As-Prepared Al_8_B_4_C_7_ Powder

**Figure 6:**
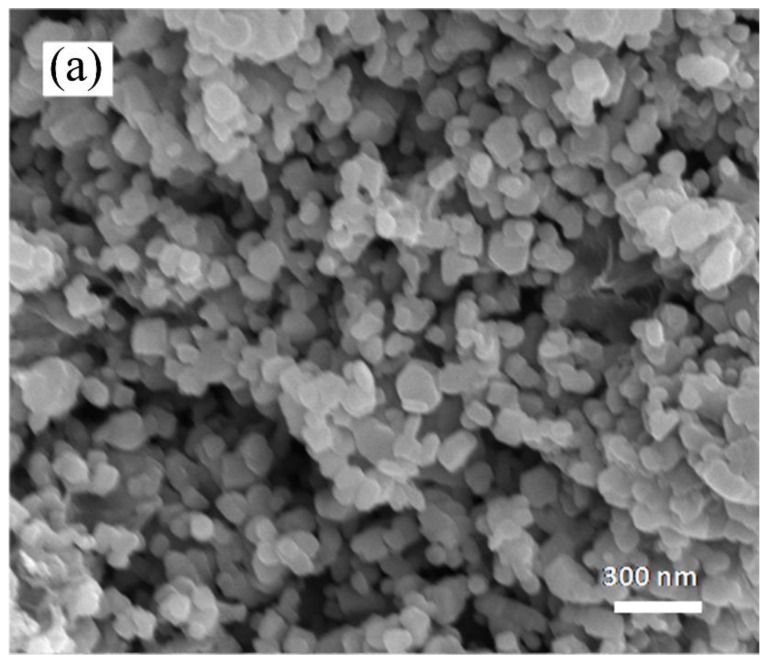

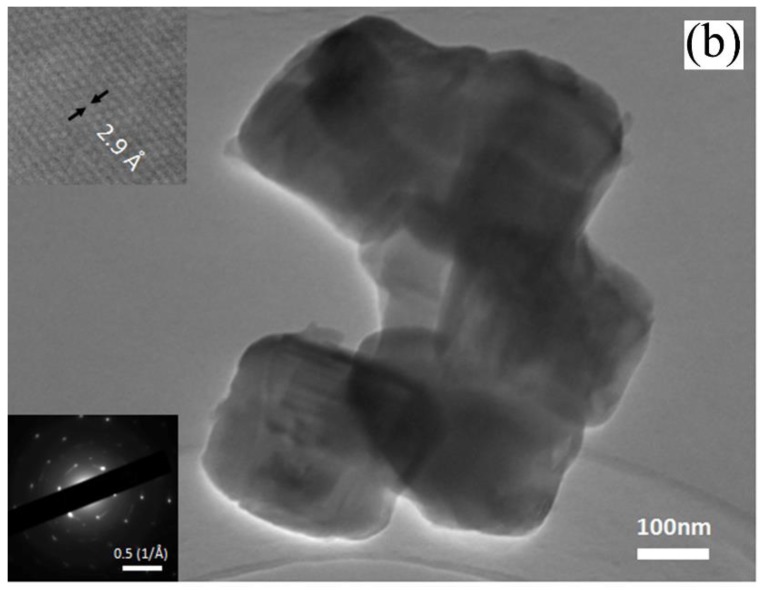
(**a**) SEM and (**b**) TEM images of Al_8_B_4_C_7_ product powder prepared in 95%NaCl + 5%NaF at 1250 °C for 6 h.

Figure 6 presents SEM and TEM images of Al_8_B_4_C_7_ particles synthesised in 95%NaCl + 5%NaF at 1250 °C for 6 h, revealing their irregular morphologies and average size of about 200 nm. The particles overall were dispersed well, though some were agglomerated together. The average size of the particles was much smaller, and their dispersion was much better than it was when the conventional synthesis techniques were used [15,16,17,18]. The lattice interlayer spacing (one of the insets in Figure 6) was measured as around 0.29 nm, which corresponds to the (111) plane of hexagonal Al_8_B_4_C_7_. This, in addition to the selected area electron diffraction (SAED) pattern (the other inset in Figure 6) and the XRD results in Figure 1 and Figure 2, verified that the synthesised particles were Al_8_B_4_C_7_.

## 4. Further Discussion and Reaction/Synthesis Mechanisms

Upon increasing the firing temperature above their melting/eutectic points, NaCl (melting point: ~714 °C) and NaF (melting point: ~743 °C) interacted with each other, forming a liquid medium in which Al slightly dissolved [26,27]. The dissolved Al diffused rapidly through the liquid medium onto the surfaces of C and B_4_C, and then reacted with them to form Al_4_C_3_, and Al_3_BC + AlB_2_, according to Equations (3) and (4), respectively.

4Al + 3C= Al_4_C_3_ (3)

9Al + 2B_4_C = 2Al_3_BC + 3AlB_2_ (4)

AlB_2_ = Al + 2 B (5)

Since AlB_2_ is not thermodynamically stable at >1000 °C [28], the AlB_2_ formed from Equation (4) decomposed, forming Al and B (Equation (5)) in the molten salt [28]. The detection of Al_4_C_3_/AlOOH indicated the occurrence of Equation (3) at the test temperatures (Figure 1a–c and Figure 2a–c), and the detection of Al_3_BC and AlB_2_ in the Al-B_4_C sample (Figure 3) indicated the occurrence of Equation (4). The AlB_2_ phase detected in this case is believed to be formed upon cooling from the Equation between the residual Al and B in the salt. The B formed from Equation (5) at the test temperatures also slightly dissolved in the molten salt [29,30] and then diffused through the molten salt onto the surface of the Al_4_C_3_ formed from Equation (3), forming Al_8_B_4_C_7_ according to Equation (6). As shown in Figure 4, Al_8_B_4_C_7_ was formed in the fired Al_4_C_3_-B-C sample, indicating that the original Al_4_C_3_ reacted directly with the B dissolved in the salt, to form Al_8_B_4_C_7_.

7Al_4_C_3_ + 12 [B] = 3Al_8_B_4_C_7_ + 4 [Al] (6)

According to Figure 3a, if no carbon was present, the intermediate Al_3_BC formed from Equation (4) appeared to be stable. However, when C was present, it was readily converted into more stable Al_8_B_4_C_7_ (Figure 4). This also explained why no Al_3_BC was found in the samples whose XRD patterns are shown in Figure 1 and Figure 2. The mechanism by which it was transformed into Al_8_B_4_C_7_ in the molten salt was not clear, but a plausible mechanism could be considered as follows: when C was present, it reacted with the Al in the molten salt to form Al_4_C_3,_ which further reacted with the B in the molten salt to form Al_8_B_4_C_7_. The consumption of Al and B in the molten salt might have led to the decomposition of Al_3_BC and thus the additional formation of Al_8_B_4_C_7_ according to Equation (7).

7Al_3_BC = 13 [Al] + Al_8_B_4_C_7_ + 3 [B] (7)

The overall reaction processes/mechanisms described above can also be used to explain the effects of firing temperature and salt type/assembly on the MSS process. With an increase in the firing temperature, the solubilities of Al and B in the molten salt were increased, and their diffusions in the molten salt accelerated. Consequently, Equations (3)–(7) were greatly facilitated. Therefore, the overall formation reaction (Equation (1)) was considerably accelerated (Figure 1, Figure 2, Figure 3 and Figure 4). When a single salt of NaCl was used, there was only limited formation of Al_8_B_4_C_7_ in the sample after 6 h of firing at 1250 °C (Figure 5a). However, when small amounts of NaF (2.5%) were added to NaCl, much more Al_8_B_4_C_7_ was formed (Figure 5b). Upon further increasing NaF to 5%, the formation reaction was completed, and essentially phase-pure Al_8_B_4_C_7_ was obtained (Figure 5c). This can be explained as follows. Al and B have very limited solubility in molten NaCl [31], so Equations (3)–(7) proceeded very slowly in it. However, when NaF was added to NaCl, the solubilities of Al and B in the binary salt were increased significantly, which led to great acceleration of Equations (3)–(7), i.e., the overall formation reaction (Equation (1)).

Thanks to the strong accelerating effect of the NaCl-NaF binary salt discussed above, essentially phase-pure Al_8_B_4_C_7_ particles were successfully prepared at 1250 °C. This synthesis temperature was 350–550 °C lower than that required by the conventional synthesis routes [11,13,14,15,16,17,18,19,20,21], demonstrating the great advantage and feasibility of the MSS technique developed in this work.

## 5. Conclusions

A low-temperature molten salt synthesis technique was developed to synthesise high-quality Al_8_B_4_C_7_ particles. The main conclusions can be drawn as follows.

1. Al_8_B_4_C_7_ particles with an average size of about 200 nm were successfully synthesised after 6 h of firing in NaCl-NaF at 1250 °C, from Al, B_4_C and C starting powders. They were essentially phase-pure and generally well-dispersed.
2. Compared with the temperature required by a conventional synthesis technique, the synthesis temperature (1250 °C) in the present work was significantly lower (350–500 °C lower), owing to the great accelerating effect of NaCl-NaF salt.
3. Al_8_B_4_C_7_ particles were formed via the following mechanisms: at the test temperatures, NaCl and NaF interacted with each other, forming a liquid medium in which Al slightly dissolved. The dissolved Al diffused rapidly through the molten salt onto the surfaces of C and B_4_C, reacting with them to form Al_4_C_3_, and Al_3_BC + AlB_2_, respectively. AlB_2_ is not stable at >1000 °C, so at the test temperatures, it decomposed into B and Al. The newly formed B also slightly dissolved in the salt, diffused onto the surface of the Al_4_C_3_ formed earlier, and reacted with it to form Al_8_B_4_C_7_, which consumed Al and B in the salt, making the Al_3_BC formed earlier decompose into additional Al_8_B_4_C_7_, Al and B.
